# Social Grooming in Bats: Are Vampire Bats Exceptional?

**DOI:** 10.1371/journal.pone.0138430

**Published:** 2015-10-07

**Authors:** Gerald Carter, Lauren Leffer

**Affiliations:** Department of Biology, University of Maryland, College Park, Maryland, United States of America; Università degli Studi di Napoli Federico II, ITALY

## Abstract

Evidence for long-term cooperative relationships comes from several social birds and mammals. Vampire bats demonstrate cooperative social bonds, and like primates, they maintain these bonds through social grooming. It is unclear, however, to what extent vampires are special among bats in this regard. We compared social grooming rates of common vampire bats *Desmodus rotundus* and four other group-living bats, *Artibeus jamaicensis*, *Carollia perspicillata*, *Eidolon helvum* and *Rousettus aegyptiacus*, under the same captive conditions of fixed association and no ectoparasites. We conducted 13 focal sampling sessions for each combination of sex and species, for a total of 1560 presence/absence observations per species. We observed evidence for social grooming in all species, but social grooming rates were on average 14 times higher in vampire bats than in other species. Self-grooming rates did not differ. Vampire bats spent 3.7% of their awake time social grooming (95% CI = 1.5–6.3%), whereas bats of the other species spent 0.1–0.5% of their awake time social grooming. Together with past data, this result supports the hypothesis that the elevated social grooming rate in the vampire bat is an adaptive trait, linked to their social bonding and unique regurgitated food sharing behavior.

## Introduction

Long-term cooperative relationships are most evident in primates [1–6], but evidence for similar social relationships has been accumulating for several other social vertebrate groups [3, 7, 8], including cetaceans [9, 10], bats [11], elephants [12], hyenas [13–15] and ravens [16–20]. The functional importance of these complex social relationships across different species may have led to similar cognitive or behavioral mechanisms for manipulating social bonds [19–24]. A prime example of such a mechanism is social grooming—the cleaning of the body by a partner. Experimental and observational studies show that primate social grooming can be ‘exchanged’ for multiple social benefits, including reciprocal grooming, social tolerance, access to food, and agonistic support [1, 25–37]. Individuals can spend up to 20% of their time grooming others [38], and the behavior provides proximate physiological rewards for both givers and receiver [39–41]. Although most of what is known about social grooming comes from studies of primates, evidence for a role of social grooming in maintaining social ties is emerging from several other mammals (marsupials [42], deer [43], cows [44], horses [45], voles [46], mice [47], meerkats [48, 49], coati [50, 51], lions [52]) and group-living birds [53, 54].

In bats, adult social grooming is female-biased in species with female philopatry [55–58], and has been most studied in the common vampire bat (*Desmodus rotundus*) [55, 59–60]. Kerth et al. [57] compared social grooming rates of vampire bats with the temperate and insectivorous Bechstein’s bat (*Myotis bechsteinii*). These two species both have long lifespans and demonstrate fission-fusion social dynamics, where individuals maintain long-term social associations while moving between several roost trees [61–63]. In both species, social grooming rates among individuals were not predicted by self-grooming or numbers of parasites [55, 57]. Bechstein’s bats spent more time grooming themselves (38% of their time in roosts) compared with vampires (23% of their roosting time), but wild vampire bats spent about 5% of their roosting time grooming others, which is 2–4 times higher than Bechstein’s bats [57, 64].

Patterns of social grooming among categories of individuals also differed between the two species. In the Bechstein’s bat, adult female social grooming was not detectably symmetrical, and was predicted by kinship, occurring mostly between adult mothers and daughters, sometimes between sisters, and only rarely between non-kin [57]. In vampires, female social grooming was highly symmetrical and relatively common among non-kin, where it correlated with co-roosting association and food sharing [55, 60].

It is not entirely clear if vampire bat social grooming is typical or exceptional when compared to other bats or non-primate mammals. One hypothesis is that social grooming in vampire bats is exceptional in quantity and quality, because it is related to their uniquely cooperative food sharing behavior [55]. Like many primates, reciprocal patterns of vampire bat food sharing and social grooming extend beyond mother-offspring bonds, suggesting they may provide both direct and indirect fitness benefits [11, 60, 65]. Among bats, the common vampire has an extraordinarily large brain and neocortex for its body size [66, 67]. In primates, increased neocortex size has been linked to higher metrics of social complexity, such as social grooming network size [68] and strategic deception [69].

Alternatively, the apparent distinctiveness of vampire bat social grooming might stem from purely ecological factors. Social grooming may be more obvious in vampire bats due to higher levels of ectoparasite infestation. Bat fly density has been linked to species-level grooming rates [70] and the two vampire species that were observed ranked 5^th^ and 6^th^ place out of 53 neotropical bats for average number of parasitic streblid flies per bat [71]. A sampling bias could also over-emphasize social grooming in vampire bats, because there is much effort focused on studying vampire bat social behavior [65] and a lack of data on social grooming in other bats.

Comparing social grooming data across studies can be difficult due to study differences in ectoparasite density, temperature, sampling method, visibility, and level of human disturbance. In this study, we took advantage of an opportunity to compare captive vampire bats with four other captive group-living bat species housed at the same facility under the same light/dark schedule, temperature, humidity, and levels of human disturbance. We compared adult-to-adult social grooming in vampire bats *Desmodus rotundus*, two frugivorous bats *Carollia perspicallata* and *Artibeus jamaicensis* (Family: Phyllostomidae) and two Paleotropical fruit bats, *Rousettus aegyptiacus* and *Eidolon helvum* (Family: Pteropodidae). Importantly, the adult bats we compared have fixed levels of social association (stable group composition) and no insect ectoparasites.

## Methods

### Animal care

All bats were cared for by the Organization for Bat Conservation (at the Cranbrook Institute of Science, Bloomfield Hills, Michigan; under permits: USDA 34-C-0117; US Fish and Wildlife Service MB003342-0), and housed at 25–28 degrees Celsius with >33% humidity on a half-reversed 12 h light/dim light cycle in flight cages that allowed free association among cagemates. Male *Artibeus*, *Carollia*, *Eidolon*, and *Rousettus* were housed together (4.5 x 3 x 2 m), while females and a few castrated males (see below) of these species were housed together in a different cage (same dimensions). All *Desmodus* were housed together (3 x 1.5 x 2 m), but sex could still be assigned with certainty because sexes tended to segregate (into female groups with a dominant male and satellite male groups) and bats were individually marked. In all cages, bats were free to hang at any place in the cage. In all species, we only observed interactions between adult bats, and only one dependent offspring was present (in *Desmodus*). Work was approved by the University of Maryland Institutional Animal Care and Use Committee (Protocol R-13-30).

### Scoring behaviors

To compare social grooming and other behaviors across species, we conducted focal sampling. We took instantaneous (“on the beep”) focal samples of a randomly chosen bat. For *Artibeus*, *Desmodus*, and *Carollia*, we obtained 13 samples from both males and females (see Table 1 for numbers of bats in each cage). For Eidolon and Rousettus, we took 13 samples from males. There was a chance (*Eidolon* = 1/3, *Rousettus* = 2/9) that attempts to sample a female bat actually sampled a castrated male, because 6 female *Eidolon* and 9 female *Rousettus* were housed with two castrated male *Eidolon* and two castrated male *Rousettus*. In Table 1, we therefore refer to this category (female and castrated male) as the “no testes” sample. Observers chose a focal bat randomly by counting bats left to right until a specific random number was reached. If a focal bat was asleep or became lost from view, the observer immediately began focal sampling the next closest bat in the same species and sex category.

**Table 1:** Means percentage of awake time spent social grooming in six bat species.

Family	Species	Number of focal samples	Mean % (and 95% confidence interval)
Phyllostomidae	*Artibeus jamaicensis*	All 26 samples	0.51 (0.06–1.09)
		13 samples from 15 females	0.90 (>0.01–1.92)
		13 samples from 21 males	0.13 (>0.01–0.38)
	*Carollia perspicillata*	All 26 samples	0.13 (>0.01–0.50)
		13 samples from 16 females	0.26 (>0.01–0.77)
		13 samples from 6 males	0.00
	*Desmodus rotundus*	All 26 samples	3.65 (1.47–6.28)
		13 samples from 15 females	5.38 (1.67–9.75)
		13 samples from 16 males	1.92 (0.13–4.87)
Pteropodidae	*Eidolon helvum*	All 26 samples	0.26 (>0.01–0.64)
		13 samples from 6 females and 2 castrated males	0.00
		13 samples from 6 males	0.51 (>0.01–1.28)
	*Rousettus aegyptiacus*	All 26 samples	0.13 (>0.01–0.32)
		13 samples from 9 females and 2 castrated males	0.26 (>0.01–0.64)
		13 samples from 5 males	0.00
Vespertilionidae	*Myotis bechsteinii*	87 samples from 8 females; data from Kerth et al. [57]	0.7

During each of 26 focal sampling sessions, observers took one observation every 10 s for 10 min (1,560 observations per species). During each observation, observers reported the presence or absence of social grooming (chewing or licking another bat’s body), self-grooming (scratching or licking its own body), feeding, and aggression. We measured self-grooming to see if social grooming differed merely due to differences in grooming, we measured feeding because feeding rates could limit and hence explain social grooming rates, and we measured aggression to see if this behavior was positively or negatively linked to social grooming. Focal sampling sessions occurred at haphazard times when bats were active. However, using logistic fit in JMP 12 we detected no effect of sample time on presence of social grooming either overall or within each species. Because observers also switched to a different species and sex after each observation, there was no reason to expect biased sampling of sexes or species.

### Statistical analysis

To calculate percent time spent social grooming, self grooming, fighting, or feeding, we divided the number of observations of these behaviors by 60 (the number of samples per session). We compared social grooming rates across both species and sexes. To test if vampire bats performed social grooming more than other species, we conducted nonparametric comparisons using the Dunn Method for joint ranking in JMP 12 with *Desmodus* as the control species. This method computes ranks on all observations then compares *Desmodus* to all other species in a pairwise manner, and provides p-values with a Bonferroni adjustment. We repeated this analysis for self-grooming. To test for an effect of sex on social grooming rates, we compared social grooming in males and ‘no testes’ bats across all species using a Wilcoxon test. To compute means and 95% confidence intervals of social grooming rates, we used bootstrapping with 1000 permutations in R [72].

To check for potential inter-observer bias, we ran a permuted linear model in R (lmPerm package) testing for effects of both ‘species’ and ‘observer’ on social grooming rates. We also repeated this procedure with ‘feeding rates’ and ‘aggression’ instead of ‘observer’ to see if observed differences in these factors could help explain social grooming rates after accounting for species differences.

## Results

**Fig 1:**
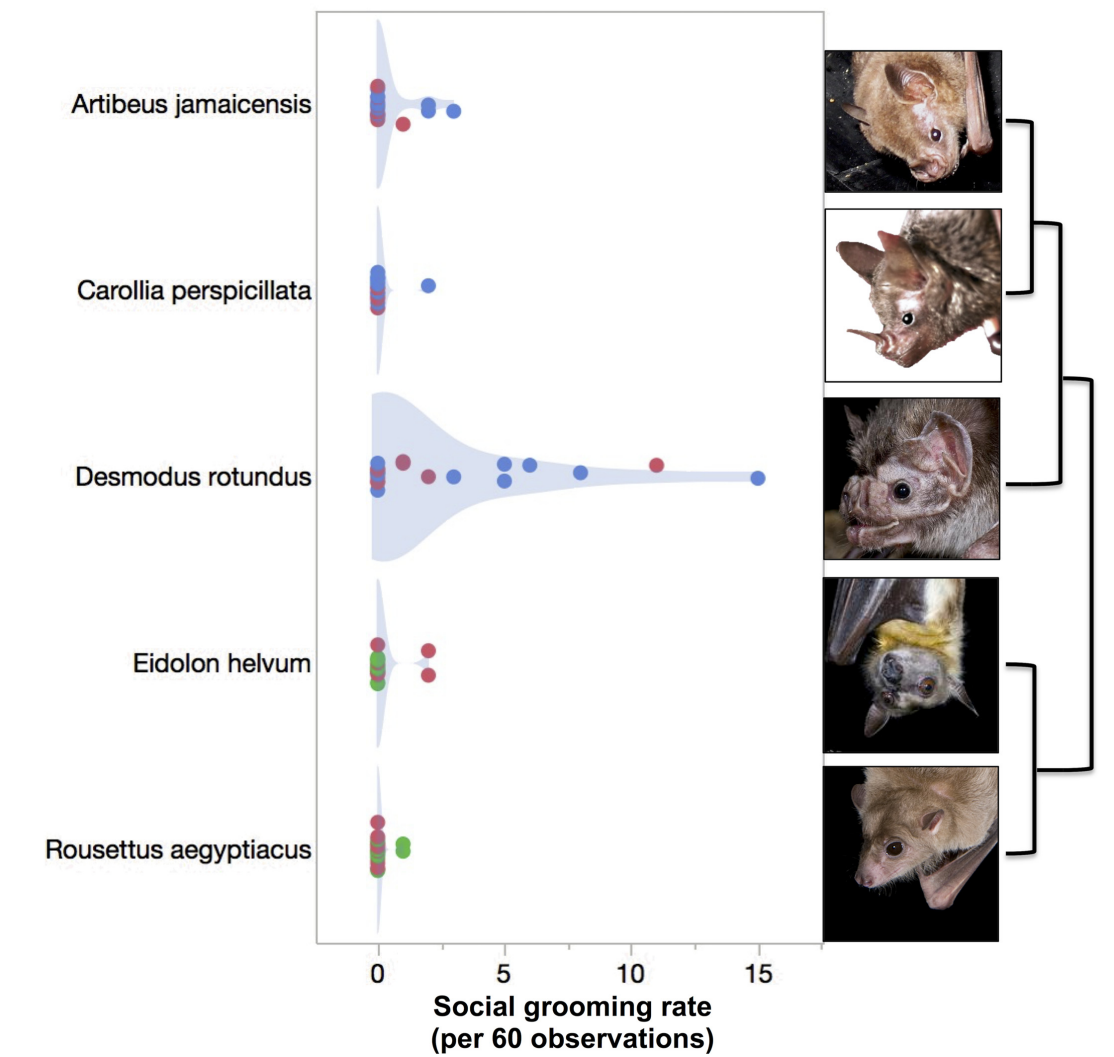
Social grooming rates in five captive bat species. Social grooming rates are shown for male (red), female (blue), and non-testes bats (green, see methods). Light blue shading shows probability density functions. Phylogenetic relationships between species are shown on right.

**Fig 2:**
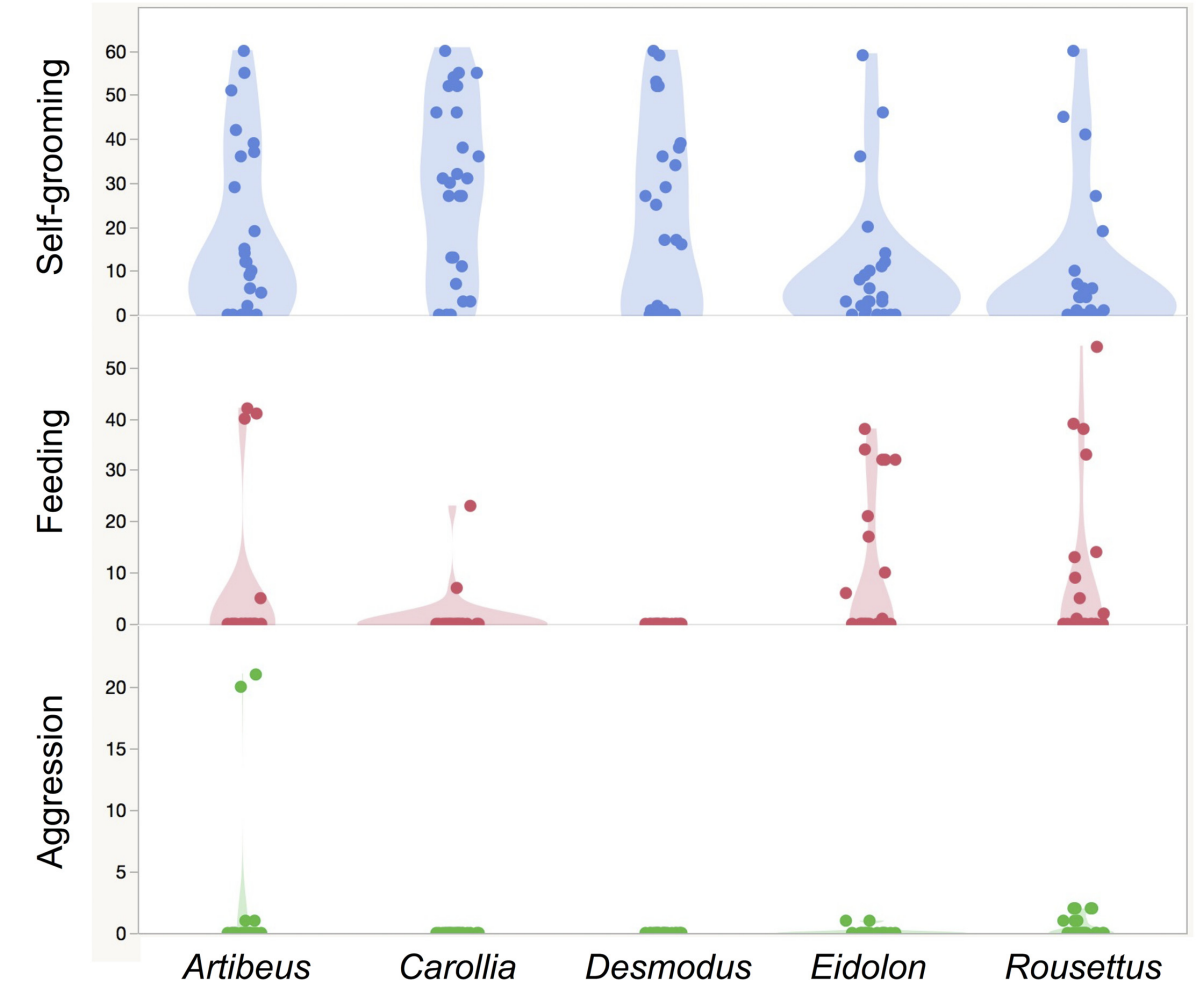
Frequency of three other behaviors in five captive bat species. Jittered dots show sample rates and shaded areas show probability density functions.

Time spent social grooming varied by species and sex (Table 1). Social grooming rates for *Desmodus* were higher than for the other species: *Artibeus* (z = 2.53, n = 26, p = 0.045), *Carollia* (z = 3.66, n = 26, p = 0.001), *Eidolon* (z = 3.28, n = 26, p = 0.004), and *Rousettus* (z = 3.35, n = 26, p = 0.003; Fig 1). In contrast, self-grooming rates in *Desmodus* were not significantly different than those for *Artibeus* (z = 0.02, p = 1), *Carollia* (z = 1.69, p = 0.36), *Eidolon* (z = 1.31, p = 0.77), and *Rousettus* (z = 2.09, p = 0.15; Fig 2). After controlling for the effect of species on social grooming rates, we detected no effect of observer (F(6, 119) = 0.19, p~1), feeding rates (F(1,124) = 0.07, p~1), or aggression (F(1,124) = 0.02, p = 0.3).

## Discussion

Under similar conditions of captivity and in the absence of external parasites, social grooming in vampire bats (*Desmodus rotundus*) is elevated beyond the rates found in four other group-living bat species (Fig 1). Vampire bats spent about 1.5–6.3% of their time social grooming, whereas time spent social grooming in the other species reached only 0.5% (Table 1). Vampires spent on average 14 times longer social grooming than the non-vampires. This difference between vampires and the other bats does not extend to self-grooming (Fig 2), and is unlikely to be explained by variation in feeding rates.

Are social grooming differences explained by general differences in social structure? The bat species in this study are all group-living in the wild, but vary in their social structures and degree of sociality. *Desmodus rotundus* forms stable female social groups of 8–12 adults with offspring, living in a roost site that is defended by a single dominant male against invasions by other males, i.e. resource defense polygyny [62, 64]. Individual recognition is evident from patterns of social behavior [64, 65], and may occur at a distance through individually variable contact calls [73], which allow vocal discrimination in another vampire bat species [74–75]. *Carollia perspicillata* female groups vary in size from 2–18 bats, and are based around resource defense polygyny with males performing courtship displays and defending territories [76, 77]. Vocal discrimination is evident between males [78] and between mothers and pups [79]. In contrast with vampire bats, group membership appears to be less stable [76]. *Artibeus jamaicensis* also demonstrates resource defense polygyny, and both males and females appear to form cooperative relationships in the wild [58, 80–82]. Dominant males are able to defend larger groups of females by tolerating the presence of subordinate males that are often kin and that help ward off foreign males [81]. These male alliances for cooperative defense of female groups can last more than 2 years [82]. Female groupmates engage in social grooming, and females that are closer to the group’s center are groomed more often [58]. Unlike vampire bats, juveniles do not appear to perform allogrooming [82]. Relatively little is known about the social behavior in *Rousettus aegyptiacus* and *Eidolon helvum* in the wild, but both species form aggregations of hundreds to thousands of both male and female individuals [83]. These aggregations likely mask the presence of smaller social networks. *Rousettus* pups converge on adult repertoire of calls through vocal learning [84]. Both species are highly vocal and frequently squabble among each other. In summary, the differences in social grooming rates we observed cannot easily be explained by straightforward differences in basic social structure.

Vampire bats are the only bats that perform regurgitated food sharing [11], and the necessity to maintain food-sharing relationships might be important to the evolution of their elevated social grooming rates. Foraging vampire bats are likely to either receive a large meal of blood or none at all, meaning that the costs of sharing are low and benefits of receiving are great [64]. Obtaining regurgitated food donations from social partners appears to be a crucial component to the inclusive fitness of vampire bats, because nearly 1/5 of bats fail to feed on a given night and individuals can starve in under 72 hours [64]. Correlational evidence suggests that vampire bats may use social grooming to maintain social bonds that are crucial for reciprocal food sharing [55,60]. Social grooming requires a relatively small investment of energy compared to regurgitated food sharing, and therefore may be used as way to build such cooperative relationships gradually with increasing investments. This ‘raising-the-stakes’ hypothesis [85] is consistent with the observation that previously unfamiliar vampire bats placed together in captivity developed social grooming but not food sharing over several weeks [65].

Wilkinson [55] suggested that social grooming might help hungry begging bats detect the ability of partners to share food, and other observations show that donors often initiate food sharing by ‘greeting’ and grooming recipients [60]. Social grooming could therefore function as a tactile signal of desire to receive food or an intention to share it. Further comparative studies on social grooming in bats and other mammals will provide insight into whether frequent social grooming may have originated from selective pressures that are similar to those having shaped social grooming in primates. Based on what is known from six bat species from three families (Table 1), the high levels of social grooming observed in vampire bats seem unlikely to reflect a general trait conserved across bats. Rather, elevated social grooming in vampire bats appears to be an adaptive specialization to a cooperative social life.
